# Physicochemical and antioxidant properties of Malaysian honeys produced by *Apis cerana*, *Apis dorsata* and *Apis mellifera*

**DOI:** 10.1186/1472-6882-13-43

**Published:** 2013-02-23

**Authors:** Mohammed Moniruzzaman, Md Ibrahim Khalil, Siti Amrah Sulaiman, Siew Hua Gan

**Affiliations:** 1 Department of Pharmacology, School of Medical Sciences, Universiti Sains Malaysia, 16150, Kubang Kerian, Kelantan, Malaysia; 2 Human Genome Centre, School of Medical Sciences, Universiti Sains Malaysia, 16150, Kubang Kerian, Kelantan, Malaysia

**Keywords:** Acacia honey, Pineapple honey, Tualang honey, Antioxidants, *Apis cerana*, *Apis dorsata*, *Apis mellifera*

## Abstract

**Background:**

The aim of the present study was to evaluate the physicochemical and antioxidant properties of Malaysian monofloral honey samples—acacia, pineapple and borneo honey—and compare them with tualang honey. Acacia and pineapple honey are produced by *Apis mellifera* bees while borneo and tualang honey are produced by *Apis cerana* and *Apis dorsata* bees, respectively.

**Methods:**

The physical parameters of honey, such as pH, moisture content, electrical conductivity (EC), total dissolved solids (TDS), color intensity, total sugar and apparent sucrose content, were measured. Hydroxymethylfurfural (HMF) was measured using high performance liquid chromatography, and a number of biochemical and antioxidant tests were performed to determine the antioxidant properties of the honey samples.

**Results:**

Acacia honey was the most acidic (pH 3.53), whereas pineapple honey had the lowest moisture content (14.86%), indicating that both types of honey can resist microbial spoilage more effectively when compared to tualang honey (pH 3.80 and 17.53% moisture content). Acacia honey contained the highest EC (0.76 mS/cm), whereas borneo honey had the highest (377 ppm) TDS. The mean HMF content in Malaysian honey was 35.98 mg/kg. Tualang honey, which is amber color, had the highest color intensity (544.33 mAU). Acacia honey is the sweetest, and contained the highest concentration of total sugar, reducing sugar and apparent sucrose. Tualang honey had the highest concentration of phenolic compounds (352.73 ± 0.81 mg galic acid/kg), flavonoids (65.65 ± 0.74 mg catechin/kg), DPPH (59.89%), FRAP values (576.91 ± 0.64 μM Fe (II)/100 g) and protein content (4.83 ± 0.02 g/kg) as well as the lowest AEAC values (244.10 ± 5.24 mg/kg), indicating its strong antioxidant properties. Proline, an important amino acid that is present in honey was also measured in the present study and it was found at the highest concentration in pineapple honey. Several strong correlations were found among the biochemical and antioxidant parameters of all the Malaysian honeys.

**Conclusion:**

Although Malaysian honeys are of good quality, tualang honey contains the strongest antioxidant properties by far.

## Background

Honey is a natural supersaturated sugar solution, which is mainly composed of a complex mixture of carbohydrates. In addition to carbohydrate content, it also contains approximately 20% water as well as minor but important constituents such as proteins, enzymes (invertase, glucose oxidase, catalase, and phosphatases), amino acids, organic acids (gluconic acid, acetic acid), lipids, vitamins (ascorbic acid, niacin, pyridoxine), volatile chemicals, phenolic acids, flavonoids, carotenoid-like substances, and minerals [1-3]. Although the composition of honey can be variable and is primarily dependent on its floral, geographical, and entomological source, certain external factors, such as seasonal and environmental factors and processing, also play important roles [4-7].

The quality of honey is determined by its sensorial, chemical, physical and microbiological characteristics [8]. The criteria that define the physicochemical quality of honey are specified by the EC Directive 2001/110 [9]. The major criteria of interest are moisture content, electrical conductivity (EC), ash content, reducing and non-reducing sugars, free acidity, diastase activity and hydroxymethylfurfural (HMF) content [1,8]. The comparative physicochemical characteristics of honeys from other regions of the world have been extensively studied [2,3,7,10-12], but there is a lack of data on Malaysian honey.

The antioxidant properties of honey have been attributed to some of the constituents present in honey. These constituents include phenolic acids and flavonoids [13], certain enzymes (glucose oxidase and catalase) [14,15], ascorbic acid, proteins and carotenoids [4]. Other reports established a correlation between floral origin and phenolic compounds and flavonoids [13,16,17]. Furthermore, the therapeutic role of honey in the treatment of various ailments has received substantial attention recently, and its therapeutic value has partly been credited to its antioxidant properties [6,18].

Malaysia, a tropical country rich with flora and fauna, harbors many different types of honey. Although tualang honey has been extensively used in the local Malaysian community for the treatment of various diseases [19], many different types of honey are consumed either directly or indirectly in many different foods in Malaysia. Among the different types of honey, the antioxidant properties of tualang and gelam honey have been previously reported [19-23]. However, this is the first time that such data for acacia and pineapple honey have been reported. Our study is also the first extensive report on the physicochemical and antioxidant properties of tualang, acacia and pineapple honeys.

Acacia honey is produced by *Apis mellifera*, a cultured bee that harvests the extrafloral nectar of *Acacia mangium* trees. Pineapple honey is also produced by the *Apis mellifera* bees that collect the nectar of pineapple (*Ananas comosus*) flowers, resulting in a honey with a distinctive aroma and flavor. It is a monofloral honey. Borneo honey is produced locally by *Apis cerana*, smaller sized local bees from the Sabah Rural Development Corporation Apiary in Kudat, East Malaysia. The bees collect the nectar mainly from *Acacia mangium* trees and flowers. It is also known as tropical honey. Tualang honey is a wild multi-floral honey produced by *Apis dorsata* bees. The bees collect nectar from plants and blossoms in the tropical rain forest in the state of Kedah in the West Cost of Peninsular Malaysia. The honey obtained its name from a tall *Koompassia excelsa* tree, known locally as the “Tualang tree,” in which the bees build their hives (Table 1).

**Table 1:** Malaysian honey, floral type and sources

**Name**	**Floral type (bee species)**	**Local (scientific) tree name**
Acacia honey	Monofloral (*Apis mellifera*)	Forest Mangrove or Mangium tree *(Acacia mangium)*
Pineapple honey	Monofloral (*Apis mellifera*)	Pineapple (*Ananas comosus)*
Borneo honey	Monofloral (*Apis cerana*)	Forest mangrove or Mangium tree *(Acacia mangium)*
Tualang honey	Multifloral (*Apis dorsata*)	Tualang tree (*Koompassia excelsa)*

The aim of the present study was to increase the currently scarce knowledge of the chemical composition of monofloral Malaysian honeys produced by different bee species as well as of their antioxidant activities.

## Methods

### Honey samples

The following four local Malaysian honey samples (acacia, pineapple, borneo and tualang) were analyzed: 1) acacia honey from *Apis mellifera* was supplied by Koperasi Alnoor from Johor Bharu; 2) pineapple honey from *Apis mellifera* was supplied by Jabatan Pertanian from Usahawan Lebah Madu Company; 3) borneo honey from *Apis cerana,* the jungle of Borneo was supplied by Koperasi; and 4) tualang honey from *Apis dorsata* was supplied by the Federal Agriculture Marketing Authority (FAMA) of Malaysia. All honey collections were conducted between July 2010 and September 2010, and the samples were refrigerated (4-5°C) in airtight plastic containers until further analysis.

### Chemicals and reagents

Ascorbic acid, bovine serum albumin (BSA), catechin, 2,2-diphenyl-1-picrylhydrazyl (DPPH), 2,4,6-tris(1-pyridyl)-1,3,5-triazine (TPTZ), HMF, Folin–Ciocalteu’s reagent, gallic acid and proline were purchased from Sigma-Aldrich (St. Louis, Mo., U.S.A.). Sodium carbonate (Na_2_CO_3_), aluminum chloride (AlCl_3_), sodium nitrite (NaNO_2_) and sodium hydroxide (NaOH) were purchased from Merck (Darmstadt, Germany). All chemicals used were of analytical grade.

### Physical analysis

#### pH

A pH meter (HI 98127, Hanna instruments, Mauritius) was used to measure the pH of a 10% (w/v) solution of honey prepared in milli-Q water (Millipore Corporation, Billerica, Massachusetts, USA).

#### Moisture content

The moisture content was determined using a refractometric method. In general, the refractive index increases with an increase in the solid content of a sample. The refractive indices of honey samples were measured at ambient temperature using an Atago handheld refractometer (KRUSS, HRH30, Hamburg, Germany), and measurements were further corrected for the standard temperature of 20°C by adding a correction factor of 0.00023/°C. The moisture content was measured in triplicate, and the percentage of moisture content that corresponds to the corrected refractive index was calculated using Wedmore’s table [24].

#### Total sugar content

Honey was suspended in milli-Q water to make a 25% (w/v) solution. The total sugar content of each honey sample was determined using a refractometric method (Atago handheld refractometer, ATAGO, N-1α, Tokyo, Japan). The percentage of sucrose content was measured per g/mL of honey.

#### Electrical conductivity and total dissolved solids

Electrical conductivity and total dissolved solids were measured using an HI 98311 conductivity meter (Hanna Instruments, Mauritius) and a 20% (w/v) solution of honey suspended in milli-Q water [25]. The electrical conductivity of the milli-Q water was determined to be less than 10 μS/cm. The electrical conductivity and total dissolved solids of each sample were analyzed in triplicate, and the mean values were expressed in mS/cm and ppm, respectively.

#### Honey color analysis

The color intensity of honey samples was measured according to the Pfund classifier. Briefly, homogeneous honey samples devoid of air bubbles were transferred into a cuvette with a 10 mm light path until the cuvette was approximately half full. The cuvette was inserted into a color photometer (HI 96785, Hanna Instruments, Cluj County, Romania). Color grades were expressed in millimeter (mm) Pfund grades when compared to an analytical-grade glycerol standard. Measurements were performed in triplicate for each sample using the approved color standards of the United States Department of Agriculture (USDA) [26].

#### Color intensity (ABS_450_)

The mean absorbance of honey samples was determined using the method of Beretta et al. [27]. Briefly, honey samples were diluted to 50% (w/v) with warm (45 - 50°C) milli-Q water, and the resulting solution was filtered using a 0.45 μm filter to remove large particles. The absorbance was measured at 450 and 720 nm using a spectrophotometer, and the difference in absorbance was expressed as mAU.

#### Determination of HMF by high-performance liquid chromatography (HPLC)

HMF concentrations were determined using an HPLC method based on the method published by the International Honey Commission (IHC) [28]. Briefly, honey samples (10 g each) were diluted to 50 mL with distilled water, filtered using a 0.45 μm nylon membrane filter and injected (20 μl) into an HPLC system (Waters 2695, Milford, MA, USA) equipped with a Photodiode Array Detector (PDA) (Waters 2996). The HPLC column used was a Merck Purospher Star RP-18e (150 × 4.6 mm, 5 μm) fitted with a guard cartridge packed with a similar stationary phase (Merck, Germany).

The HPLC method included an isocratic mobile phase of 90% water and 10% methanol with a flow rate of 1.0 mL/min. All solvents used were of HPLC grade. The detection wavelength was 200–450 nm, with specific monitoring at 285 nm. The HMF concentrations in the samples were calculated by comparing the corresponding peak areas of the sample and HMF standard solutions (Sigma-Aldrich, USA) after correcting for the dilution of honey samples. A linear relationship (r^2^ = 0.9997) was determined between the concentration and the area of HMF peaks (results are expressed in mg/kg).

### Analysis of antioxidant properties

#### Determination of total phenolic compounds

The concentration of phenolic compounds in honey samples was estimated using a modified spectrophotometric Folin-Ciocalteu method [29]. Briefly, 1 mL of honey extract was mixed with 1 mL of Folin and Ciocalteu’s phenol reagent. After 3 min, 1 mL of 10% Na_2_CO_3_ solution was added to the mixture and adjusted to 10 mL with distilled water. The reaction was kept in the dark for 90 min, after which the absorbance was read at 725 nm using a T 60 UV/VIS spectrophotometer (PG Instruments Ltd, UK). Gallic acid was used to calculate a standard curve (20, 40, 60, 80 and 100 μg/mL; r^2^ = 0.9970). The concentration of phenolic compounds was measured in triplicate. The results were reported as the mean ± standard deviation and expressed as mg of gallic acid equivalents (GAEs) per kg of honey.

#### Determination of total flavonoid content

The total flavonoid content in each honey sample was measured using the colorimetric assay developed by Zhishen et al. [30]. Honey extract (1 mL) was mixed with 4 mL of distilled water. At the baseline, 0.3 mL of NaNO_2_ (5%, w/v) was added. After five min, 0.3 mL of AlCl_3_ (10% w/v) was added, followed by the addition of 2 mL of NaOH (1 M) 6 min later. The volume was then increased to 10 mL by the addition of 2.4 mL distilled water. The mixture was vigorously shaken to ensure adequate mixing, and the absorbance was read at 510 nm. A calibration curve was created using a standard solution of catechin (20, 40, 60, 80 and 100 μg/mL; r^2^ = 0.9880). The results were expressed as mg catechin equivalents (CEQ) per kg of honey.

#### DPPH free radical-scavenging activity

The antioxidant properties of each honey sample were also studied by evaluating the free radical-scavenging activity of the DPPH radical, which was based on the method proposed by Ferreira et al. [31]. Briefly, honey extract (0.5 mL) was mixed with 2.7 mL of methanolic solution containing DPPH radicals (0.024 mg/mL). The mixture was vigorously shaken and left to stand for 15 min in the dark (until their absorbance stabilized). The reduction of the DPPH radical was determined by measuring the absorbance of the mixture at 517 nm (Hatano *et al.*, 1988).

Butylated hydroxytoluene (BHT) was used as a reference. The radical-scavenging activity (RSA) was calculated as the percentage of DPPH discoloration using the following equation: % RSA = ([A_DPPH_– A_S_]/A_DPPH_) × 100, where A_S_ is the absorbance of the solution when the sample extract has been added at a particular level and A_DPPH_ is the absorbance of the DPPH solution.

#### Ferric reducing-antioxidant power assay (FRAP assay)

The FRAP assay was performed according to a modified method described by Benzie and Strain [32]. Briefly, 200 μL of properly diluted honey (0.1 g/mL) was mixed with 1.5 mL of FRAP reagent. Then, the reaction mixture was incubated at 37°C for 4 min, and its absorbance was read at 593 nm against a blank that was prepared with distilled water. Fresh FRAP reagent was prepared by mixing 10 volumes of 300 mM/L acetate buffer (pH 3.6) with 1 volume of 10 mM TPTZ solution in 40 mM/L HCl containing 1 volume of 20 mM ferric chloride (FeCl_3_.6H_2_O). The resulting mixture was then pre-warmed at 37°C. A calibration curve was prepared using an aqueous solution of ferrous sulfate (FeSO_4_.7H_2_O) at 100, 200, 400, 600 and 1000 μM/L. FRAP values were expressed as micromoles of ferrous equivalent (μM Fe [II]) per kg of honey.

#### Determination of ascorbic acid content

The ascorbic acid content was measured using the method described by Ferreira et al. [31]. Briefly, the sample (100 mg) was extracted with 10 mL of 1% metaphosphoric acid at room temperature for 45 min and filtered through Whatman No. 4 filter paper. The filtrate (1 mL) was mixed with 9 mL of 0.005% 2,6-dichlorophenolindophenol (DCPIP), and the absorbance of the mixture was measured within 30 min at 515 nm against a blank. The ascorbic acid content was calculated based on a calibration curve of authentic L-ascorbic acid (50, 100, 200 and 400 μg/mL; Y = 3.2453X - 0.0703; r^*2*^ = 0.9440). The results were expressed as mg of ascorbic acid per kg of honey.

#### Antioxidant content

The antioxidant content was determined by measuring AEAC (Ascorbic acid Equivalent Antioxidant Capacity) values using the method of Meda et al. [13]. Briefly, honey samples were dissolved in methanol to a final concentration of 0.03 g/mL. A 0.75 mL aliquot of the methanolic honey solution was then mixed with 1.50 mL of a 0.02 mg/mL DPPH solution prepared in methanol. The mixture was incubated at room temperature for 15 min, and the absorbance was measured at 517 nm using a spectrophotometer. The blank was composed of 0.75 mL of a methanolic honey solution mixed with 1.5 mL of methanol. Ascorbic acid standard solutions (1, 2, 4, 6 and 8 μg/mL) prepared in milli-Q water were used to form a calibration curve. Measurements were performed in triplicate, and the mean value was expressed as mg of ascorbic acid equivalent antioxidant content per 100 g of honey.

#### Proline content

The proline content in honey samples was measured using a method established by the IHC [33]. Briefly, BSA solutions were prepared by diluting a stock solution of 1 mg/mL as appropriate and final concentrations ranged from 0.05 to 1.00 mg/mL. From the dilutions, 0.2 mL of the protein solution was transferred to different test tubes, and 2 mL of alkaline copper sulfate reagent (analytical reagent) was added, followed by thorough mixing. The resulting solution was incubated at room temperature for 10 min. Then, 0.2 mL of Folin Ciocalteau solution was added to each tube and incubated for 30 min. The absorbance was measured at 660 nm.

### Biochemical analyses

#### Protein content

The protein content of the honey was measured according to Lowry’s method [33]. Briefly, BSA solutions were prepared by diluting a stock solution of 1 mg/mL as appropriate and final concentrations ranged from 0.05 to 1.00 mg/mL. dilutions, 0.2 mL of protein solution was placed in different test tubes, and 2 mL of alkaline copper sulfate reagent (analytical reagent) was added. After the resulting solution was mixed properly, it was incubated at room temperature for 10 min. Then, 0.2 mL of reagent Folin Ciocalteau solution was added to each tube and incubated for 30 min. The colorimeter was calibrated with a blank, and the absorbance was measured at 660 nm.

#### Reducing sugar assay

The total reducing sugar content was measured using 3,5-dinitrosalicylic acid (DNSA). In principle, the reducing sugar reduces DNSA to 3-amino-5-nitrosalicylic acid, resulting in a solution with reddish-orange coloration that is measured spectrophotometrically at 540 nm [2]. The honey solution (0.1 g/mL) was diluted 100-fold with milli-Q water. A 1 mL aliquot of this diluted solution was mixed with equal amounts of DNSA solution and incubated in a boiling water bath for 10 min. The mixture was allowed to cool to ambient temperature for 10 min and was then mixed with 7.5 mL of milli-Q water; then, the absorbance was measured at 540 nm using a spectrophotometer. A glucose solution of known concentrations (100, 200, 400 and 600 μg/mL) was used as a standard.

#### Statistical analysis

Assays were performed in triplicate, and the results were expressed as mean values with standard deviations (SD). The significant differences, represented by letters, were obtained by a one-way analysis of variance (ANOVA), followed by Tukey’s honestly significant difference (HSD) post hoc test (p < 0.05). Correlations were established using Pearson’s correlation coefficient (r) in bivariate linear correlations (p < 0.01). These correlations were calculated using Microsoft Office Excel 2007 and SPSS version 16.0 (IBM corporation, New York, USA).

## Results and discussion

### Physical analyses

#### pH and moisture content

All of the investigated Malaysian honey samples were acidic (pH 3.53 - 4.03) (Table 2) and were within the limit (pH 3.4 to 6.1) that indicates freshness. Among all the honey types, acacia honey was the most acidic (pH 3.53 ± 0.06). The pH values of Malaysian honey samples were similar to those previously reported in Indian, Algerian, Brazilian, Spanish and Turkish honeys (between pH 3.49 and 4.70) [2,10,12,34]. The high acidity of honey is an indication of the fermentation of sugars present in the honey into organic acid, which is responsible for two important characteristics of honey: flavor and stability against microbial spoilage [35].

**Table 2:** Physical parameters (pH, moisture content, electrical conductivity, total dissolved solids concentrations and color intensity) of Malaysian honey samples

**Sample**	**pH**	**Moisture content (%) mean ± SD**	**EC (mS/cm) mean ± SD**	**TDS (ppm) mean ± SD**	**HMF (mg/kg) mean ± SD**	**ABS**_**450 **_**(mAU; 50 w/v) mean ± SD**
Acacia *(A. mellifera)*	3.53 ± 0.06^c^	15.16 ± 0.10^c^	0.76 ± 0.005^a^	375.00 ± 2.5^a^	0.26 ± 0.2^d^	320.33 ± 2.8^b^
Pineapple *(A. mellifera)*	3.73 ± 0.06^b^	14.86 ± 0.20^c^	0.35 ± 0.002^b^	176.00 ± 1.0^c^	68.99 ± 0.44^a^	312.33 ± 12.34^b^
Borneo *(A. cerana)*	4.03 ± 0.06^a^	16.99 ± 0.31^b^	0.75 ± 0.006^a^	377.00 ± 2.0^a^	28.50 ± 1.05^c^	338.33 ± 17.10^b^
Tualang *(A. dorsata)*	3.80 ± 0.0^b^	17.53 ± 0.12^a^	0.75 ± 0.003^a^	371.00 ± 1.3^b^	46.17 ± 1.59^b^	544.33 ± 11.68^a^
**Mean ± SD**	3.78 ± 0.21	16.14 ± 1.33	0.65 ± 0.20	324.75 ± 99.0	35.98 ± 0.77	378.83 ± 1110.87

Means were compared by using a one-way ANOVA with post hoc multiple comparisons. In each column, values with different letters (superscripts) indicate significant differences (*p <* 0.05).

Moisture content is an important parameter of honey quality and defines the amount of water present in honey. In the present study, the percentage moisture content was between 14.86 and 17.53%, which is under the limit of ≤20% set by the international regulations for honey quality [9,36] (Table 2). There were significant differences in the moisture content between the acacia and pineapple honey samples when compared with tualang and borneo honey (one-way ANOVA; *p<* 0.05). Tualang honey had the highest moisture content (17.53%), whereas pineapple honey had the lowest moisture content (14.86%), indicating that pineapple honey is the most resistant to microbial growth because water is an essential component for microbial growth.

Generally, the moisture contents for Malaysian honeys were lower than those of other honeys, such as Portuguese honey (15.9-17.2%) [37], Anatolian honey (17.0-19.4%) [38]), Romanian honey (15.4-20.0%) [39] and Indian honey (17.2-21.6%) [2]. The moisture content present in honey samples is important as it contributes to its ability to resist fermentation and granulation during storage [40]. Low moisture content also helps to promote longer shelf life during storage [41]. Overall, the low moisture content in our honey samples indicates their good storage ability and quality.

#### Electrical conductivity (EC) and total dissolved solids (TDS)

EC is one of the important factors in the determination of the physical characteristics of honey [42]. The EC values of all Malaysian honey samples were 0.35-0.76 mS/cm (Table 2) and were within the allowed parameters (lower than 0.8 mS/cm) set by Codex Alimentarius [36]. Acacia honey, which showed the highest EC value (0.76 mS/cm), contained the highest amount of minerals, as opposed to pineapple honey, which had the lowest EC value (0.35 mS/cm). Both tualang and borneo honey showed similar EC values, indicating the presence of similar amounts of minerals. Furthermore, the EC value changes when the amount of plant pollen decreases [43]. Overall, the Malaysian honey samples had similar EC values to those reported for honey samples from Uruguay [44]; Andalusia, Spain [42]; India [2]; and Morocco [41].

TDS assesses the combined content of both inorganic and organic substances present in honey, including molecular, ionized and micro-granular suspended forms. In our study, borneo honey exhibited the highest TDS value (377 ppm), whereas pineapple honey showed the lowest (176 ppm). There was a positive correlation between the EC and TDS values; for example, pineapple honey, which showed the lowest EC value, had the lowest TDS value.

#### Total sugar content

The mean total sugar content of Malaysian honey samples was 65.53 ± 2.48% g/mL of honey (Table 3). None of the samples exceeded the highest limit for the total sugar content of honeys established by the European community directive [9]. Our result is similar to that reported for some of the Algerian honeys (62-70%) [3] and honey samples from Bangladesh (42.80 to 60.67%) [45]. The high sugar content of the investigated honey samples can be attributed to its high acidity and low moisture content, which inhibits the formation of HMF from sugars, especially glucose and fructose. The highest total sugar content (68.40%) was from acacia honey, indicating its high natural sweetness, which was confirmed when the honey was physically tasted. Indian [2] and Estonian honey samples [46] reportedly have relatively higher total sugar contents (78.4-82.4% and 62.88- 78.32%).

**Table 3:** Reducing and non-reducing sugar content of Malaysian honey samples

**Sample**	**Total sugar content (g/100 g honey)**	**Reducing sugar (g/100 g honey)**	**Apparent sucrose (g/100 g honey)**
	**mean ± SD**	**mean ± SD**	**mean ± SD**
Acacia *(A. mellifera)*	68.40 ± 0.80^a^	63.89 ± 0.25^a^	4.51 ± 1.05^a^
Pineapple *(A. mellifera)*	63.33 ± 0.92^b^	61.17 ± 0.17^b^	2.17 ± 0.94^b^
Borneo *(A. cerana)*	66.80 ± 0.80^a^	63.06 ± 0.54^a^	3.74 ± 1.03^a^
Tualang *(A. dorsata)*	63.60 ± 0.80^b^	61.94 ± 0.75^b^	1.66 ± 0.73^b^
**Mean ± SD**	65.53 ± 2.48	62.51 ± 1.20	3.02 ± 1.33

Means were compared using a one way ANOVA with post hoc multiple comparisons. In each column, values with different letters (superscripts) indicate significant differences (*p <* 0.05).

#### Color characteristics

Color is the primary characteristic for honey classification according to the USDA-approved color standards [26]. Honey’s color naturally varies over a wide range of tones, ranging from light yellow to amber, dark amber and, in extreme cases, it may be black. Occasionally, even green or red hues may occur [3,45]. The color of untreated honey depends on its botanical origins. For this reason, color is very important in the classification of monofloral honeys for commercial activities.

**Figure 1:**
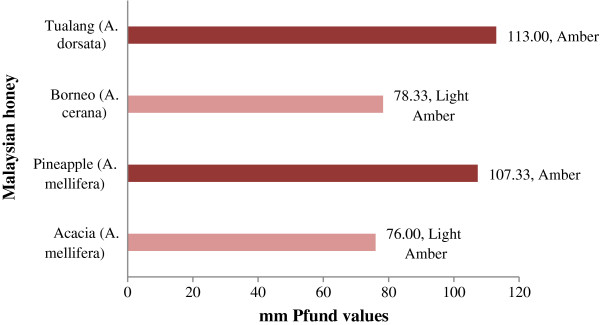
Color characteristics of Malaysian honey.

In the present study, tualang honey is classified as amber according to the USDA-approved color standards [26], and it also exhibited the highest Pfund value (113.00) (Figure 1), which was similar to the mean Pfund value reported for Algerian honey samples (114.00) [3] but was lower than that reported for Bangladeshi honey samples [45]. However, acacia and borneo honey are light amber in color and have lower Pfund values. The higher Pfund values may indicate a higher antioxidant potential and the presence of different pigment compounds, such as phenolic compounds, flavonoids and carotenoids.

#### Color intensity ABS_450_

The color intensity of the honey is represented by the ABS_450_, which also indicates the presence of pigments such as carotenoids and flavonoids, which are known to have antioxidant properties [36]. In the present study, ABS_450_ values ranged from 312 to 544 mAU (Table 2). Tualang honey, which had the highest Pfund values, also showed the highest color intensity (544.33 ± 11.68 mAU). Previously reported Pfund values for tualang honey were also high (489.5 ± 1.7 mAU) [19]. The color intensity of the acacia, pineapple and borneo honeys was much lower (312.33 - 338.33 mAU), indicating lower antioxidant properties. To our knowledge, these are the first reported Pfund values for acacia, pineapple and borneo honeys.

#### Determination of HMF concentrations by HPLC

HMF is an important parameter used to indicate the purity and freshness of honey [47]. HMF is usually present in trace amounts in fresh honeys, but its levels tend to increase during processing and/or due to aging. For example, in a previous study conducted by Khalil et al. [47], the HMF level was reported to be very high in Malaysian tualang honey, ranging from 128.19–1131.76 mg/kg when the honeys were stored for more than one year. HMF levels are also influenced by several other factors, such as pH, temperature, duration of the heating process, storage conditions and floral source; therefore, HMF levels provide an indication of overheating and poor storage conditions [48].

With the exception of pineapple honey, which contained a relatively high HMF concentration (68.99 mg/kg), the HMF concentrations of honey samples ranged from 0.26 to 46.17 mg/kg (Table 2). However, all values were still within the recommended range (<80 mg/kg) set by the Codex Alimentarius [36]. The higher HMF concentration in pineapple honey may also be due to the type of sugar content as well as its fructose:glucose ratio [49]. Some Australian honeys, namely rainforest, homebrand and mallee honey, were reported to have HMF concentrations of 2.2, 17.7 and 34.0 mg/kg, respectively [48,50].

### Antioxidant properties

#### Total phenolics compounds

The mean total phenolics compounds of the tested honeys was 243.01± 74.91 mg gallic acid/kg. The total phenolic compounds is sensitive to phenol and polyphenol entities and other electron-donating antioxidants such as ascorbic acid and vitamin E. The phenolic compound in Malaysian honeys varied greatly depending on the type of honey. Tualang honey contained the highest level (352.73 ± 0.81 mg/kg) (Table 4). These variations may be due to the different floral sources of the honey analyzed. The levels of phenolic compounds of tualang honey in this study was higher than that previously reported for tualang honey (251.7 ± 7.9 mg gallic acid/kg) [19], but lower than that reported for some Burkina Fasan honey (74.38 ± 20.54 mg gallic acid/100 g) [13] and Manuka honey (52*.*63 ± 1*.*21 mg gallic acid/100 g) [22]. This indicates that both Burkina Fasan and Manuka honeys have higher antioxidant potential when compared to tualang and other Malaysian honey samples.

**Table 4:** Biochemical and antioxidant properties of Malaysian honey samples

**Sample**	**Total phenolics mean ± SD (mg galic acid/kg)**	**Total flavonoids mean ± SD (mg catechin/kg)**	**FRAP values mean ± SD (μM Fe (II)/100 g)**	**Proline mean ± SD (mg/kg)**	**Protein mean ± SD (g/kg)**
Acacia *(A. mellifera)*	186.70 ± 0.84^d^	21.95 ± 1.73^d^	100.90 ± 2.44^c^	517.55 ± 1.48^b^	2.04 ± 0.01^d^
Pineapple *(A. mellifera)*	226.29 ± 1.18^b^	37.39 ± 0.90^b^	87.47 ± 1.10^d^	628.69 ± 3.75^a^	2.69 ± 0.01^b^
Borneo *(A. cerana)*	206.33 ± 1.05^c^	25.81 ± 0.64^c^	256.64 ± 0.60^b^	176.64 ± 2.31^d^	2.16 ± 0.02^c^
Tualang *(A. dorsata)*	352.73 ± 0.81^a^	65.65 ± 0.74^a^	576.91 ± 0.64^a^	248.53 ± 1.33^c^	4.83 ± 0.02^a^
**Mean±SD**	243.01 ± 74.91	37.70 ± 19.75	255.48 ± 227.63	392.85 ± 215.05	2.93 ± 1.30

Means were compared by using one-way ANOVA with post hoc multiple comparisons. In each column, values with different letters (superscripts) indicate significant differences (*p <* 0.05).

#### Total flavonoid content

Flavonoids are low-molecular-weight phenolic compounds that affect the aroma and antioxidant properties of honey. The mean flavonoid content of the Malaysian honey samples was 37.70 ± 19.75 mg catechin/kg (Table 4). As with phenolic compounds, the honey samples showed significant differences in flavonoid content. Similar to the polyphenol content, tualang honey contained the highest amount (65.65 mg/kg) of flavonoids. The flavonoid content in acacia honey was lower (21.95 mg/kg) when compared to Croatian acacia honey (43.66 mg/kg) [51] and Burkina Fasan acacia honey (61.4 mg/kg) [13]. This could be due to the different floral and geographical origins of the honey sources.

Generally, the flavonoid content of Malaysian honeys is lower than that reported for some Algerian honeys (27 to 71 mg/kg) [3] but higher than that reported for Linen vine honey (25.2 mg/kg); Christmas vine honey (10.9 mg/kg) [4]; eucalyptus honey (20–25 mg CE/kg); sunflower and rape honey (15–20 mg CE/kg); and fir, lavender, ivy and acacia honey (5–10 mg CE/kg), as previously reported [13,31]. The variations in the flavonoid levels could be due to the different honey types and their sources. It has been suggested that measuring phenolic compounds and flavonoids levels could be used to study honey’s floral and geographical origins [17].

#### DPPH free radical-scavenging activity

DPPH is an unwavering nitrogen-centered radical that has been extensively used to test the free radical scavenging ability of various samples. In evaluating the radical-scavenging potential of honeys, the DPPH assay is frequently used because the antioxidant potential of honey has been shown to be directly associated with its phenolic acid and flavonoid content [27], where high DPPH scavenging activity confers superior antioxidant activity.

**Figure 2:**
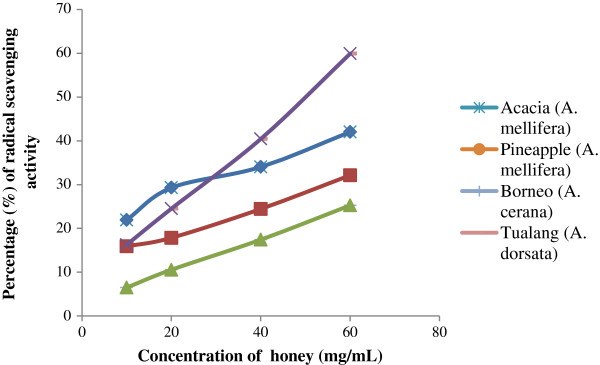
Percentage of inhibition of DPPH radical scavenging activity at different concentrations of Malaysian honey.

When the DPPH radical scavenging activities of all honey samples were measured at 10, 20, 40 and 60 mg/mL, the highest percentage of inhibition was observed at 60 mg/mL for all of the samples. Tualang honey exhibited the highest percentage inhibition (59.89%), again indicating that it has the highest antioxidant potential (Figure 2). The percentage of inhibition shown by tualang honey in this study is higher than what was previously reported for tualang honey (41.30%) [19] and also higher than that reported for Indian honey samples (57.5%) and Algerian honey samples (44.55%) [3]. In the present study, the higher concentrations of phenolic compounds and flavonoids present may have been responsible for the higher percentage of radical-scavenging activity shown.

#### Determination of total antioxidant content by FRAP assay

FRAP is a simple, direct test widely used for the determination of antioxidant activity in many different substances, including honey [1,18,27,32,38,52]. It gives a direct estimation of the antioxidants or reductants present in a sample based on its ability to reduce the ferric to ferrous (Fe^3+^/Fe^2+^) couple.

The mean FRAP value of Malaysian honey samples was 255.48 ± 227.63 μM Fe [II]/100 g. Again, tualang honey exhibited the highest FRAP value (576.91 ± 0.64 μM Fe (II)/100 g) (Table 4). Our reported FRAP value for tualang honey is higher than that reported for Algerian honeys (287.45 to 403.54 μM Fe (II)/100 g) [3]; (322.1 ± 9.7 μM Fe [II]/100 g) [19] but lower than that reported by Khalil et al. [22] for tualang honey (706*.*91 ±7*.*28 μM Fe [II]/100 g). The higher FRAP values of tualang honey may be due to its stronger antioxidant properties compared with all the other Malaysian honey samples, indicating a greater reduction of Fe^3+^ to Fe^2+^ ions corresponding to samples with a higher reducing power that increased in absorbance at 700 nm. The lowest FRAP value (87.47 μM Fe [II]/100 g) was exhibited by pineapple honey, indicating its low antioxidant potential.

#### Ascorbic acid content

**Figure 3:**
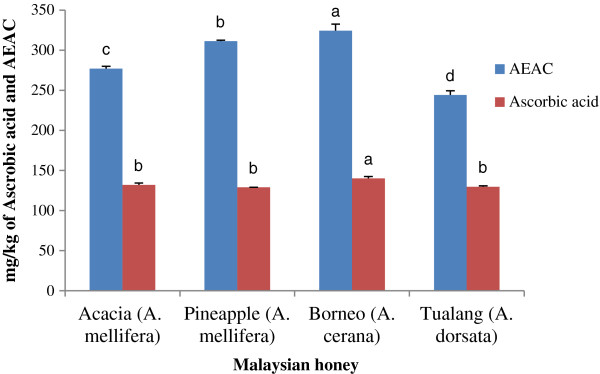
**Ascorbic acid and AEAC (Ascorbic acid Equivalent Antioxidant Capacity) contents of Malaysian honey.** Values with different letters indicate significant differences (*p <* 0.05).

In addition to polyphenols, honey contains a number of compounds known to act as antioxidants, including ascorbic acid and enzymes such as glucose oxidase and catalase [4]. Malaysian honey samples have ascorbic acid levels ranging from 128.98 to 140.14 mg/kg (Figure 3). These values are slightly lower than those reported for Portuguese honey samples (140 to 145 mg/kg) [31] and Algerian honey samples (156 to 164 mg/kg) [3].

These variations could be due to having samples from different geographical regions and floral sources as well as differences in storage time. When honey is stored for a longer duration, the concentrations of several other compounds may also decrease, which can affect the levels of not only ascorbic acid but also enzymes [53]. In the present study, borneo honey showed the highest ascorbic acid content (140.14 ± 2.18 mg/kg). This may be attributed to the presence of a higher amount of ascorbic acid in *Acacia mangium* itself, which needs to be further investigated.

#### Ascorbic acid Equivalent Antioxidant Capacity (AEAC) assay

The AEAC content of Malaysian honey samples was measured in mg of AEAC/100 g of honey using an ascorbic acid standard curve (r^2^ = 0.9787). The samples exhibited AEAC values ranging from 276.96 to 324.47 mg of AEAC/kg (Figure 3). These values are slightly higher than those reported in Indian (between 151 and 295 mg of AEAC/kg) [2], Burkina Fasan (270.40 ± 146.8 mg/kg) [13] and Algerian (236 mg/kg to 315 mg/kg) [3] honey samples. These results indicate that Malaysian honey samples also have a high antioxidant potential. To our knowledge, this is the first report on the ascorbic acid content and AEAC values of Malaysian honey samples. Because a lower AEAC value indicates stronger antioxidant properties, tualang honey, which had the lowest AEAC value (244.10 mg/kg), may contain the strongest antioxidant properties.

#### Proline content

Proline is an important amino acid that is produced mostly from the salivary secretions of bees during the conversion of nectar into honey [54]. Proline levels are dependent on the type of flower that the bees visit and therefore may differ based on the floral source. Proline content is a sign of honey ripeness, and it is postulated that honeys with a high proline content have a lower probability of being adulterated with sugar [55].

The mean proline concentration of Malaysian honey was 392.85 ± 215.05 mg/kg. To our knowledge, this is the first study to report the proline levels of acacia, borneo and pineapple honeys. Pineapple honey contained the highest proline concentration (628.69 ± 3.75 mg/kg), possibly due to the higher amino acid content in pineapple or *ananas comosus* itself, which should be further investigated.

Our results indicate that the proline content in Malaysian honey samples is similar to that in Indian (133–674 mg/kg) [2], Algerian (202–680 mg/kg) [12] and Bangladeshi honey (106–681 mg/kg) [45]. The higher amount of proline in all of the analyzed Malaysian honey samples indicates the honey’s ripeness and that there is less probability for sugar adulteration, which confirms that the honey samples analyzed are of good quality.

### Biochemical analyses

#### Reducing sugar content

Our data indicate that reducing sugars are the main soluble sugars present in Malaysian honey because the total reducing sugar content in the samples was as high as 61.17 to 63.89% (Table 3). To our knowledge, this is the first data reported for the total sugar, reducing sugar and sucrose contents in Malaysian honey. The EC Directive 2001/110 mandates that the amount of reducing sugars should be ≥60 g/100 g of honey, with the exception of honeydew honey, which has a lower allowable limit (≥45 g/100 g). Thus, our results meet this standard and are similar to other published levels of reducing sugars [2,3,37].

The amount of non-reducing sugars, including the sucrose content (%), was measured by subtracting the amount of reducing sugars present from the total sugar content. The sucrose content in Malaysian honey samples ranged from 1.66 to 4.51%, which is below 5.00%, the maximum prescribed limit set by the Codex standard [36]. Our results show that Malaysian honey samples generally have higher non-reducing sugar content than Algerian honeys (1.80 to 2.54%) [3]. As with the total sugar content, acacia honey contained the highest reducing sugar (63.89%) and sucrose content (4.51%), which contributes to its physical sweetness when compared to other types of Malaysian honey.

#### Protein content

The concentrations of proteins and amino acids in honeys differ based on their botanical or geographical origin and storage time. Enzymes are the main protein constituents present in honey [56]. The bees also add different enzymes during the honey ripening process, which can contribute to increased protein levels. In general, the protein content of honey ranges from 2 to 5 g/kg [55]. The protein content in Malaysian honeys ranged from 2.04 to 4.83 g/kg (Table 4). To our knowledge, this is the first report regarding the protein content of Malaysian honey samples.

Tualang honey also contained the highest amount of protein (4.83 ± 0.02 g/kg), which was slightly higher than that of some Algerian honeys [3]. However, in another study, relatively higher protein levels (3.7 to 9.4 g/kg) were reported in Algerian honey samples [12], whereas for honey samples from India, the content was reported to be lower (0.48 to 2.29 g/kg) [2]. This could be due to differences in the floral source as well as the geographical origins of the honey.

#### Correlation amongst biochemical parameters and antioxidant properties

Several significant correlations between biochemical and antioxidant parameters are shown in Table 5. A strong correlation was found between the color intensity of the honey samples and their antioxidant parameters, phenolic compounds, flavonoids, FRAP values and protein contents, at correlation coefficients of 0.960, 0.915, 0.964, and 0.953 respectively. This indicates that color pigments may have a role in the observed antioxidant activities of Malaysian honey samples.

**Table 5:** **Correlation matrix showing the interrelation among phenolics, flavonoids, DPPH scavenging, Ferric reducing-antioxidant power assay (FRAP), ascorbic acid, proline, Absorbance at 450 nm (ABS**_**450**_**) and protein**

	**Phenolics**	**Flavonoids**	**DPPH**	**FRAP**	**Ascorbic acid**	**Proline**	**ABS**_**450**_	**Protein**
Phenolics	1.000	0.989**	0.804**	0.914**	0.411	0.390	0.960**	0.997**
Flavonoids	0.989**	1.000	0.782**	0.848**	0.512	0.274	0.915**	0.991**
DPPH	0.804**	0.848**	1.000	0.712**	0.598*	0.131	0.840**	0.834**
FRAP	0.914**	0.848**	0.712**	1.000	0.056	0.719**	0.964**	0.889**
Ascorbic acid	0.411	0.512	0.598*	0.056	1.000	0.621*	0.267	0.471
Proline	0.390	0.274	0.131	0.719**	0.621*	1.000	0.530	0.329
ABS_450_	0.960**	0.915**	0.840**	0.964**	0.267	0.530	1.000	0.953**
Protein	0.997**	0.991**	0.834**	0.889**	0.471	0.329	0.953**	1.000

**Correlation is significant at the 0.01 level (2-tailed); *Correlation is significant at the 0.05 level (2-tailed).

Color intensity also increases with the increase in phenolic compounds and flavonoid content of honey. A strong correlation between the ABS_450_ and FRAP values suggests the involvement of pigments that grant the antioxidant properties to honey. The correlation coefficient between ABS_450_ and FRAP values was 0.83 in Indian honeys [2], whereas in some Slovenian honeys, it was 0.85 [5]. Thus, the higher correlation in our study indicates that Malaysian honeys have a stronger antioxidant capacity compared to Indian and Slovenian honeys.

Phenolic compounds and flavonoids are the most important determinants for the antioxidant properties of honey. A strong correlation exists between phenolic compounds and FRAP values (r=0.914). The correlation value of our study was higher than that for Algerian honey (r=0.668) [3] but similar to that for Indian honey (r=0.900) [2]. In addition, the correlation coefficient between the total flavonoids and FRAP values was 0.848, which is slightly lower than that of Algerian honey (r=0.893).

A significant positive linear correlation was observed between phenolic compounds and flavonoid content with DPPH and FRAP values (r=0.804, 0.782 and r=0.712), indicating that these are good indicators for antioxidant activities and that both phenolic compounds and flavonoids contribute to their radical scavenging activity.

Proline is an important amino acid that also contributes to the antioxidant properties of honey, and it was found to strongly correlate with FRAP and ascorbic acid content. The most significant correlation was observed between the proline content and FRAP values (r=0.721), which is similar to the result reported for some Indian honeys (r=0.73) [2], indicating that the proline content also contributes to the antioxidant potential of Malaysian honey.

Protein content was also strongly correlated with phenolic compounds (r=0.997), flavonoids (r=0.991), FRAP (r=0.889) and ABS_450_ (r=0.953). Ascorbic acid, an important vitamin that is well known for its antioxidant properties, was also significantly correlated with the proline content (r=0.621), indicating that proline may contribute to antioxidant activities to some extent.

The correlation analysis clearly demonstrates that the overall antioxidant property in the investigated Malaysian honeys can be attributed to various factors, including phenolic compounds, flavonoids, proline and ascorbic acid contents and color pigments. Overall, phenolic compounds and flavonoid content are significant determinants of the antioxidant capacity of honey samples as well as their reducing ability and radical scavenging potential. Furthermore, phenolic compounds and flavonoid content appear to be highly important for antioxidant activity, as shown by their correlation values. Several batches of tualang honey should be studied to further confirm these findings in future.

## Conclusion

This is the first extensive investigation of the physicochemical and antioxidant properties of honeys from different botanical and entomological origin of Malaysian. This study showed that Malaysian honeys have good antioxidant potential. Among the four different honey types, tualang honey had the highest phenolic compound and flavonoid contents with the highest ferric reducing power values as well as the greatest color intensity, indicating that it has the highest antioxidant potential. Acacia honey was the most acidic and contained the highest total sugar, reducing sugar and apparent sucrose contents and the highest mineral content. Pineapple honey had the highest concentration of proline, whereas borneo honey had the highest concentration of total dissolved solids. Our study is the first to extensively report on the chemical composition and antioxidant activities of four monofloral Malaysian honeys.

## Competing interests

There is no conflict of interest statement among the authors.

## Authors’ contributions

MM carried out the experimental parts of this investigation and prepared the manuscript. MIK helped to conduct the study. SAS, and SHG supervised the work, evaluated the results and corrected the manuscript for publication. All authors read and approved the final manuscript.

## Pre-publication history

The pre-publication history for this paper can be accessed here:

http://www.biomedcentral.com/1472-6882/13/43/prepub
